# Multicentre real‐world evaluation of the Haematuria Cancer Risk Score to risk‐stratify the detection of bladder cancer in patients referred from primary care

**DOI:** 10.1002/bco2.70233

**Published:** 2026-05-29

**Authors:** Tom Malik, Jonathan Denfhy, Hpone Theinkha Lin, Lubna Mohammed, Lin Aung Han, Kirthana Babureddy, Amber Pankhurst, Edward Tudor, Amina Buba, Laura Waley, Aashna Bali, Gemma Shaw, Edward Jefferies, Jessica Head, Tirion Smith, Jade Brown, Neil Trent, Sian Dudley, Sunil Mathur, Jonathan Aning

**Affiliations:** ^1^ Bristol Urological Institute, North Bristol NHS Trust Bristol UK; ^2^ Population Health Sciences, Bristol Medical School University of Bristol Bristol UK; ^3^ Department of Urology Gloucestershire Hospitals NHS Foundation Trust Cheltenham UK; ^4^ Department of Urology Royal United Hospitals NHS Foundation Trust Bath UK; ^5^ Department of Urology Somerset Foundation Trust Taunton UK; ^6^ Department of Urology Great Western Hospitals NHS Foundation Trust Swindon UK

**Keywords:** bladder cancer, haematuria, radical cystectomy, transurethral resection of bladder tumour

## Abstract

**Objectives:**

To investigate the potential of the Haematuria Cancer Risk Score (HCRS) to improve the real‐world investigation pathway for suspected bladder cancer.

**Materials and methods:**

Data were retrospectively analysed for all consecutive patients referred with suspected urinary tract cancer on a faster diagnostic pathway to five UK institutions between January and April 2025. The HCRS cut‐off score of ≥82 was used to define a ‘HCRS high risk’ population. The co‐primary outcomes were the ability to calculate HCRS in the referred population from the information provided by primary care and the cancer detection rate.

**Results:**

In total, 1944 referrals were received, median age of 71 years (IQR 61–78), 1186/1944 (61%) were male, and 1586/1944 (82%) had sufficient information to calculate the HCRS. Of the cohort with HCRS scores, overall 165/1586 (10%) had bladder cancer. The HCRS was ≥82 in 176/437 (40%) of those with non‐visible haematuria (NVH); in total, 6/176 (3%) had bladder cancer; and using HCRS in the NVH group alone, no case of muscle‐invasive bladder cancer (MIBC) would have been missed. The HCRS was ≥82 in 1062/1149 (92%) with visible haematuria (VH), of whom 150/1062 (14%) had bladder cancer. Adopting a strategy of using HCRS and upper tract imaging in combination for the whole cohort would have resulted in two cases of NMIBC being missed for the NVH cohort and one case of NMIBC being missed for the VH cohort. No cases of MIBC or upper tract urothelial cancer would have been missed.

**Conclusion:**

HCRS is a simple innovation, which demonstrates clear potential when combined with upper tract imaging to improve current UK risk stratification to determine which patients referred with haematuria need flexible cystoscopy.

## 1 INTRODUCTION

Haematuria is the most common symptom leading to referral to investigate whether a urinary tract cancer is present. UK observational studies of referred patients with visible haematuria (VH) and non‐visible haematuria (NVH) have found urinary tract cancer detection rates of between 14% and 26% for patients with VH and 3%–6% for patients with NVH respectively.^1^, ^2^, ^3^ In recent years, there has been wide variation in recommended haematuria referral criteria internationally, based on risk.

Current National Institute for Health and Care Excellence guidelines for referral of patients with suspected bladder cancer (NICE NG12) recommend urgent referral of all patients aged 45 years and over with unexplained visible haematuria without urinary tract infection (UTI) or visible haematuria that persists or recurs despite successful treatment of UTI.^4^ NG12 also recommends referral of patients aged 60 and over with unexplained non‐visible haematuria and either dysuria or raised white cell count on blood tests. In the only prospective UK study to date that we are aware of, NG12 guidance has been demonstrated to miss urinary tract cancers and result in significant over investigation.^2^ This multicentre study was subject to the limitation that recruitment was achieved by sampling clinics and not consecutive recruitment over a specified time period. Tan et al. subsequently developed the Haematuria Cancer Risk Score (HCRS). The HCRS involves a simple calculation using weighted points for age, sex, nature of haematuria and smoking status.^5^ A HCRS score of <82 indicates low risk of malignancy, whereas a score of ≥82 highlights patients at high risk for malignant disease. Real‐world data are needed to validate these findings in other UK populations. Furthermore, bladder cancer survival rates remain stable in England.^6^ One reason for this is that all patients with haematuria are managed on the same pathway with the same investigations irrespective of risk. There is a need to test risk stratification strategies that can be used in clinical practice to improve present guidance.^7^, ^8^

The aim of this multicentre study was to evaluate the real‐world potential of HCRS to improve risk stratification of the present suspected urinary tract cancer referral pathway.

## 2 MATERIALS AND METHODS

A multicentre retrospective study was conducted of all consecutive patients referred on the faster diagnostic pathway for suspected bladder cancer, between 1 January 2025 and 30 April 2025, at five hospitals (North Bristol NHS Trust, Royal United Hospitals Bath NHS Trust, Great Western Hospitals NHS Foundation Trust, Gloucestershire Hospitals NHS Foundation Trust, Somerset Hospitals NHS Foundation Trust) in the south‐west region of England. The study was approved by the institutional review boards at each unit.

The co‐primary outcome was cancer detection rate for the three urological malignancies targeted by this pathway (bladder cancer, upper tract urothelial cancer and renal cancer) and the number of patients in whom the HCRS could be interpreted. The HCRS was calculated by addition of weighted points^5^: age = points, female = 0, male = 10.5, NVH = 0, VH = 24.5, non‐smoker = 0, ex‐smoker = 7.5, smoker = 17.1. A score <82 was considered low risk of malignancy, and a score ≥82 represented high risk. The information used to calculate the HCRS was obtained from the standard urgent suspected cancer referral form completed in primary care. Cancer detection was defined as histologically proven urothelial cancer, radiological diagnosis of upper tract urothelial cancer or radiological diagnosis of a renal tumour.

Secondary outcomes included effect of the HCRS on cancer detection rate, incidence of cancers detected by imaging alone including bladder cancers, renal tumours or upper tract urothelial cancer (UTUC) diagnosed radiologically on upper urinary tract imaging. An analysis of timelines from referral to critical time points in the bladder cancer pathway and an exploratory analysis of modelling of HCRS potential impact to improve timings and affect clinical decision making were performed. Median and interquartile range were utilised to summarise continuous variables, and the Mann–Whitney *U* test was selected for statistical comparison due to the data demonstrating a skewed distribution.

## 3 RESULTS

### 3.1 Cohort demographic

A total of 1944 patients were referred during the study period, of whom 1861/1944 (96%) underwent flexible cystoscopy and 1879/1944 (97%) had upper tract imaging. Of those referred, 1186/1944 (61%) were male, median age was 71 years (IQR 61–78), and 1731/1944 (89%) met NG12 referral criteria. The primary referral symptom was VH in 1368/1944 (70%) and NVH in 512/1944 (26%). Other features leading to referral including lower urinary tract symptoms, dysuria, UTIs or an incidental radiological finding were present in 64/1944 (3%). The cohort demographic characteristics are summarised in Table 1. Of those referred, 286/1944 (15%) underwent a general anaesthetic cystoscopy and biopsy or transurethral resection of bladder tumour (TURBT) after suspicious findings at their flexible cystoscopy and/or upper tract imaging.

**TABLE 1:** Baseline demographic data.

Characteristic	Total cohort (*n* = 1944)	Referred with VH (*n* = 1368)	Referred with NVH (*n* = 512)	Referred with other features (*n* = 64)
Age Median (IQR)	71 (61–78)	71 (60–78)	71 (63–79)	71 (66–78)
Gender				
Male	1186 (61%)	961 (70%)	191 (37%)	34 (53%)
Female	758 (39%)	407 (30%)	321 (63%)	30 (47%)
Referral compliance with NG12				
Yes	1731 (89%)	1274 (93%)	415 (81%)	42 (66%)
No	213 (11%)	94 (7%)	97 (19%)	22 (34%)
Clinician performing flexible cystoscopy				
Consultant	439 (23%)	333 (24%)	82 (16%)	25 (39%)
Resident	1394 (72%)	939 (69%)	420 (82%)	35 (55%)
Allied health professional	95 (5%)	83 (6%)	9 (2%)	3 (5%)
GP with a special interest	5 (0.3%)	4 (0.3%)	1 (0.2%)	0
Not done	11 (0.6%)	10 (0.7%)	0	1 (2%)
Upper tract imaging				
CT	1222 (63%)	1062 (78%)	127 (25%)	33 (52%)
USS	655 (34%)	260 (19%)	368 (72%)	27 (42%)
MR urogram	2 (0.1%)	2 (0.1%)	0	0
Not done	65 (3%)	44 (3%)	17 (3%)	4 (6%)
Had TURBT or GA cystoscopic bladder biopsy	286 (15%)	238 (17%)	33 (6%)	15 (23%)
HCRS				
Calculated	1586 (82%)	1149 (84%)	437 (85%)	0
Low risk (<82)	348 (22%)	87 (8%)	261 (60%)	0
High risk (≥82)	1238 (78%)	1062 (92%)	176 (40%)	0

Abbreviations: CT, computed tomography; GA, general anaesthetic; HCRS, Haematuria Cancer Risk Score; IQR, interquartile range; MR, magnetic resonance; TURBT, transurethral resection of bladder tumour; USS, ultrasound scan.

### 3.2 Cohort cancer detection rate

**FIGURE 1:**
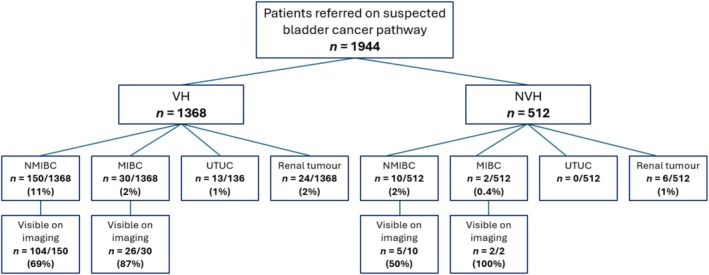
Flow diagram of malignant diagnoses for patients referred with haematuria. MIBC, muscle invasive bladder cancer; NMIBC, non‐muscle‐invasive bladder cancer; NVH, non‐visible haematuria; UTUC, upper tract urothelial cancer; VH, visible haematuria.

The cancer detection rate using standard investigations in the cohort was 257/1944 (13%). Figure 1 illustrates cancer diagnoses classified by symptoms leading to referral. Following TURBT or bladder biopsy, NMIBC was diagnosed in 169/1944 (9%) and MIBC in 33/1944 (2%). Upper tract imaging was reported for 198/202 (98%) patients diagnosed with bladder cancer. In one case without imaging available, TURBT was performed for an incidental tumour identified during gynaecological surgery, following which the patient was referred to Urology in their local region. In another case, there was no documented rationale for not performing imaging, and the remaining two patients without imaging were 85 years old with significant frailty. Among patients diagnosed with bladder cancer who underwent upper urinary tract imaging, 146/197 (74%) had evidence of a bladder tumour reported on imaging. Across the cohort, 39/1944 (2%) had renal tumours and 16/1944 (0.8%) UTUC diagnosed. Of the 1368 patients referred with VH, 150/1368 (11%) were diagnosed with NMIBC, 30/1368 (2%) with MIBC, 24/1368 (1.8%) with a renal tumour and 13/1368 (1%) with UTUC. For patients referred with NVH, 10/512 (2%) had NMIBC, 2/512 (0.4%) had MIBC, 6/512 (1%) had a renal tumour, and there were zero cases of UTUC.

Of the referrals compliant with NICE guidelines, 163/1731 (9%) were diagnosed with NMIBC, 31/1731 (2%) with MIBC, 37/1731 (2%) with a renal tumour and 15/1731 (0.9%) with UTUC. Of the 207/1944 (11%) cases with non‐compliant referrals, 6/207 (3%) had NMIBC, 2/207 (1%) had MIBC, 2/207 (1%) had a renal tumour, and 1/207 (0.5%) had UTUC. The grade and stage of the tumours diagnosed are displayed in Table S1.

### 3.3 Sub‐analyses of HCRS cancer detection rates

Complete data were available to calculate the HCRS for 1586/1944 (82%), of whom 1421/1586 (90%) met NG12 referral criteria. The reason that HCRS could not be calculated was lack of information about smoking status in 307/358 (86%) or being referred with symptoms other than haematuria in 51/358 (14%). A summary of malignant diagnoses classified by HCRS is illustrated in Table 2.

**TABLE 2:** Sub‐group analysis of cancer detection rate for patients for whom HCRS could be calculated.

Diagnosis	Low risk (HCRS <82) *n* = 348	High risk (HCRS ≥82) *n* = 1238
Total bladder cancer	9/348 (0.3%)	156/1238 (13%)
Low‐grade NMIBC	5/348 (1%)	57/1238 (5%)
High‐grade NMIBC	2/348 (0.6%)	80/1238 (6%)
T2–4 N0 M0 MIBC	1/348 (0.3%)	16/1238 (1.3%)
Metastatic MIBC	1/348 (0.3%)	3/1238 (0.2%)

Abbreviations: MIBC, muscle‐invasive bladder cancer; NMIBC, non‐muscle‐invasive bladder cancer.

In patients meeting NG12 criteria referred with NVH, the cancer detection rate in the HCRS high‐risk group and low‐risk group was 6/176 (3%) and 10/261 (4%), respectively. In patients meeting NG12 criteria referred with VH, the cancer detection rate in the HCRS high‐risk group and low‐risk group was 181/1062 (17%) and 5/87 (6%), respectively. Of all patients meeting NG12 referral criteria, 7/1731 (0.4%) tumours were not identified by HCRS high risk classification alone, though four of these were demonstrated on upper tract imaging studies. Two of the remaining three undiagnosed tumours were found to be low‐grade NMIBC on histology, and the final tumour was high‐grade NMIBC.

If only the HCRS and upper tract imaging had been performed for patients referred with NVH, 258/437 (59%) patients would have avoided flexible cystoscopy due to their HCRS being <82 and no bladder tumours being identified on imaging. This strategy would have missed bladder cancers in 2/258 (0.8%) compared to the current standard (NG12), which would have missed 2/97 (2%) patients referred with NVH who did not fulfil the referral criteria. Of the two missed cancers, one was low‐grade NMIBC, and the other was high‐grade NMIBC.

If only the HCRS and upper tract imaging had been performed for VH, 84/1149 (0.7%) patients would have avoided flexible cystoscopy due to HCRS being <82 and no bladder tumours being identified on imaging. This strategy would have missed 1/84 (1%) bladder tumour compared to the current standard (NG12), which would have missed 5/94 (5%) patients referred with VH who did not fulfil the referral criteria. This patient was diagnosed with low‐grade NMIBC on histology.

We explored the potential utility of HCRS in the scenario where a red patch was identified in the bladder at flexible cystoscopy. In this cohort, *n* = 81/1944 (4%), 30/81 (37%) were referred with NVH, and 49/81 (60%) were referred with VH. Of these patients, 3/81 (4%) were diagnosed with NMIBC, and none had MIBC. If HCRS had been applied and only those with high‐risk HCRS scores had undergone cystoscopy + biopsy, 24/65 (37%) flexible cystoscopies would have been avoided, and zero cancers would have been missed.

### 3.4 Pathway timelines to cancer diagnosis

**FIGURE 2:**
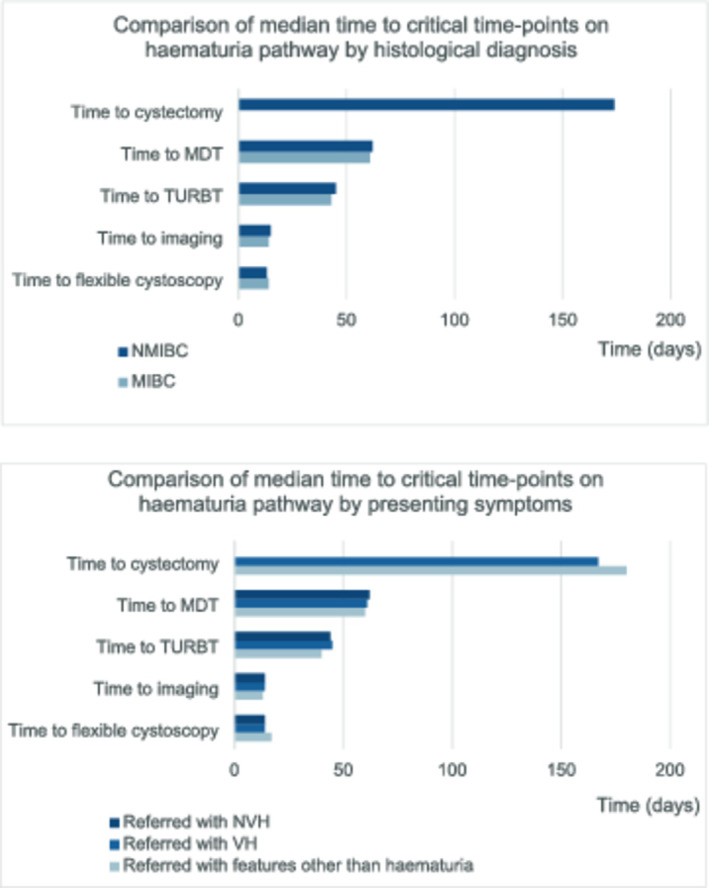
Swimmer plots of pathway timelines to diagnosis and treatment. MDT, multidisciplinary team meeting; MIBC, muscle‐invasive bladder cancer; NMIBC, non‐muscle‐invasive bladder cancer; TURBT, transurethral resection of bladder tumour.

Median time from referral to TURBT was 44 days (IQR 35–56) for the cohort, and a total of 33/1944 (2%) patients were diagnosed with MIBC. Among these, median age was 78 (IQR 68–83), 26/33 (79%) were male, and visible haematuria was the presenting complaint in 30/33 (91%). For the MIBC sub‐group, median time from referral to flexible cystoscopy was 14 days (IQR 11–25), to TURBT was 43 days (IQR 36–51) and to radical cystectomy (*n* = 8/33) was 174 days (IQR 135–186). In the cohort of 169/1944 (9%) diagnosed with NMIBC, the median time from referral to flexible cystoscopy was 13 days (IQR 10–21) and to subsequent TURBT or cystoscopic bladder biopsy was 45 days (IQR 35–56). These times are compared and contrasted with patients referred with VH, NVH or other symptoms in the swimmer plots in Figure 2.

On Mann–Whitney *U* test, there was no significant difference between patients referred with NVH or VH in time from referral to flexible cystoscopy (*p* = 0.76), to imaging (*p* = 1.00), to TURBT (*p* = 1.00) or to MDT (*p* = 1.00). Mann–Whitney *U* test also demonstrated no significant difference in speed of flow to these time points between patients found to have NMIBC or MIBC (*p* = 1.00). Patients undergoing radical cystectomy were not included in this analysis as none were referred with NVH or had NMIBC.

## 4 DISCUSSION

In a large contemporary UK real‐world population with similar cancer detection rates to previous UK observational studies, we have demonstrated that it should be feasible to implement the HCRS utilising current primary care referral information. In our study, most cases where it was not possible to calculate HCRS were because of missing data about smoking status, which could easily be addressed by gaining this information directly from patients. The present study indicates that utilising HCRS in conjunction with upper tract imaging studies to risk stratify patients referred with suspected urinary tract cancers would reduce over‐investigation using flexible cystoscopy by a fifth and miss fewer cancers than the present NG12 referral criteria. We found no significant difference between pathway times to TURBT for patients referred with NVH versus VH or between those found to have MIBC versus NMIBC, and we hypothesise that this may be contributing to the present situation in the United Kingdom where bladder cancer outcomes remain unchanged over time.^6^, ^9^

The UK real‐world population findings in this study further support Tan et al.'s external validation of the HCRS, which found flexible cystoscopy could be avoided in 25% of their study sample.^5^ Turan et al. have recently investigated whether a higher HCRS score is more predictive of patients having MIBC and concluded that HCRS used together with imaging may support risk stratification in haematuria pathways by flagging patients at higher risk of MIBC.^10^ In our cohort, the median time from referral to radical cystectomy was 174 days, which is considerably longer than the European Association of Urology (EAU)‐recommended target of within 3 months of diagnosis.^11^ We believe that our work highlights the need for continued innovation in the pathway, not only to triage suspected bladder cancer referrals but also to incorporate risk stratification more smartly to improve bladder cancer outcomes. The BladderPath study recently demonstrated that the use of magnetic resonance imaging (MRI) in the diagnostic pathway for patients with suspected MIBC could achieve a significant reduction in time to definitive treatment of 45 days.^12^ Our study illustrates the potential for reduction in the number of flexible cystoscopies performed by utilising HCRS which may result in more timely access to investigations for patients who most need them, in addition to health economic benefit through rationalisation of health system resources and more environmentally sustainable pathways; we believe that this pathway innovation merits prospective evaluation to define the benefits and harms of change at scale.^13^

Patients have identified research to investigate methods to reduce pain and discomfort during flexible cystoscopy as a high research priority.^14^ In a prospective study of UK patients from 52 centres, 77.5% of 213 participants reported ≥1 adverse event and the self‐reported prevalence of dysuria/LUTs and UTI requiring antibiotics after cystoscopy was 46.9%, 67.1% and 23.1%, respectively.^15^ We hope that our data will help support shared decision making with patients, in particular for frail or anxious patients wishing to avoid the consequences of an invasive investigation. Internationally, there are a range of different positions with regard to the investigation of haematuria. Fundamentally though guidelines differ with regard to age thresholds and more specifically NVH definitions. In contrast to the United Kingdom, the American Urological Association (AUA) explicitly states that NVH should not be defined by positive dipstick testing alone and that a positive urine dipstick test should prompt formal microscopic evaluation.^16^ The updated AUA 2025 guidelines have incorporated risk stratification and counselling of patients regarding investigations.^16^ Low‐risk patients may be considered for repeat urinalysis within 6months rather than proceeding directly to flexible cystoscopy or imaging. By contrast, Swedish national policy has abandoned investigation of asymptomatic NVH since 1999. Malstrom et al. demonstrated no adverse effect on survival following this policy and suggested potential for significant cost savings at national level as a result.^17^ Based on our analysis, we suggest that the HCRS should be considered for implementation in the United Kingdom to further refine NICE NG12 referral guidance.

We have demonstrated that, without risk stratification, patient flow through the suspected bladder cancer pathway is no quicker for those referred with VH compared to NVH or those diagnosed with MIBC compared to NMIBC. There is scope to improve time to definitive treatment, which is particularly vital for patients with MIBC. The HCRS requires four simple datapoints to calculate and inform whether a flexible cystoscopy should be performed. Other risk calculators have been designed with the aim of improving the diagnostic pathway for patients with suspected bladder cancer, most recently the IDENTIFY Urinary Tract Cancer Prediction Calculator, which uses 13 data points to substratify patients into very low, low, intermediate and high risk categories.^3^, ^18^, ^19^ The IDENTIFY risk calculator has been externally validated in an international prospective cohort where microscopy was not required to define haematuria.^20^ Khadhouri et al. reported a decision curve analysis showing a greater net benefit of using the IDENTIFY predictive model over the HCRS model to investigate patients with haematuria, compared with investigating all or none, particularly in the IDENTIFY intermediate‐ and high‐risk groups.^20^ A limitation of this study was that the histological type of cancer was not collected in the validation cohort, and therefore, they were unable to perform a subanalysis on urothelial cancers alone. Though our study was retrospective, we illustrated that the HCRS is feasible to use to risk stratify patient pathways at the point of referral before them needing to attend a secondary care facility using present real‐world UK primary care referral information, and we included the histological information of bladder tumours identified. In contrast, the predictors used in the IDENTIFY model are extensive and must be collected prospectively to ensure no missing variables, and we share the view of Khadouri et al. that at present the IDENTIFY risk calculator is a tool that is best used at present in the haematuria clinic by a healthcare professional with the patient present or in a dedicated triage remote consultation to make shared decisions rather than at the point of referral.^8^, ^20^ Both HCRS and IDENTIFY offer potential to improve and refine haematuria pathways of the future. We hope that our work contributes to informing future much needed mixed methods prospective studies to improve the haematuria patient pathway.

The present study has several limitations: Its retrospective design means that it cannot be inferred whether there would be any downstream significant risks or consequences from missing bladder cancers using an HCRS and upper tract imaging only risk stratified pathway, though our data suggest that the risks of missing a cancer are lower using a HCRS and imaging pathway than the current NG12 pathway if adhered to. We acknowledge that our findings are most likely to be applicable to the UK setting and may have limited generalisability to other healthcare systems recommending formal microscopic evaluation of urine. Our study aimed to evaluate the real‐world potential of risk stratification of patients at the point of referral from primary care. We were unable to compare the performance of HCRS with other risk stratification methods such as the IDENTIFY model because the information in referral forms from primary care was not sufficient to perform calculations. Specifically, the datapoints are as follows: Family history of urothelial cancer and whether there was a history of a single UTI/recurrent UTI/catheter/pelvic radiotherapy/suprapubic pain were seldom documented. While such comparisons may have provided useful insights, our work highlights the need to review referral data requirements, recognising practical constraints in primary care, and to work collaboratively to address these gaps. It should be noted that our study represents a secondary care population and therefore may be subject to selection bias with inclusion of higher‐risk patients; for this reason, the findings should not at present be applied to the general primary care population. On balance, therefore, while the authors believe an innovated pathway incorporating HCRS would be safe, particularly in the NVH population, an appropriately designed mixed methods prospective study could answer the question about which patients can be de‐escalated from investigations addressing the limitations of the present study. It is also important to acknowledge this study does not address or inform how to best investigate people with clinical symptoms that do not include haematuria.

## 5 CONCLUSIONS

This real‐world study has demonstrated the potential of HCRS used in combination with routinely performed imaging modalities to improve risk stratification of patients referred to secondary care with suspected bladder cancer. We have highlighted shortcomings in the speed with which patients with MIBC are investigated and treated in the present pathway and opportunities for improvement. Prospective evaluation of HCRS as a cost‐effective method to improve haematuria risk stratification is essential to define its optimal role.

## AUTHOR CONTRIBUTIONS

Tom Malik and Jonathan Aning had full access to all the data in the study and take full responsibility for the integrity of the data and the accuracy of the data analysis. *Study concept and design*: Jonathan Aning. *Acquisition of data*: Tom Malik, Jonathan Denfhy, Hpone Theinkha Lin, Lubna Mohammed, Lin Aung Han, Kirthana Babureddy, Amber Pankhurst, Amina Buba, Laura Waley, Jessica Head, Sian Dudley. *Analysis and interpretation of data*: Tom Malik and Jonathan Aning. *Drafting of the manuscript*: Tom Malik and Jonathan Aning. *Critical revision of the manuscript for important intellectual content*: Jonathan Denfhy, Hpone Theinkha Lin, Lubna Mohammed, Lin Aung Han, Kirthana Babureddy, Amber Pankhurst, Edward Tudor, Amina Buba, Laura Waley, Aashna Bali, Gemma Shaw, Edward Jefferies, Jessica Head, Tirion Smith, Jade Brown, Neil Trent, Sian Dudley and Sunil Mathur. *Supervision*: Jonathan Aning.

## CONFLICT OF INTEREST STATEMENT

Tom Malik, Jonathan Denfhy, Hpone Theinkha Lin, Lubna Mohammed, Lin Aung Han, Kirthana Babureddy, Amber Pankhurst, Edward Tudor, Amina Buba, Laura Waley, Aashna Bali, Gemma Shaw, Edward Jefferies Jessica Head, Tirion Smith, Jade Brown, Neil Trent, Sian Dudley and Sunil Mathur declare no conflicts of interest. Jonathan Aning has received research funding from Nonacus and honoraria from Astellas, AstraZeneca, Bayer, J&J Innovative Medicine, Merck and Nonacus.

## ETHICS STATEMENT

Ethics approval was not applied for or required for this study. The study was performed in accordance with the Declaration of Helsinki.

## Supporting information


**Table S1:** Grade and stage of bladder tumours.

## Data Availability

Data supporting the results reported in the article can be made available upon written request to the authors.
