# Supplementary material for: Multicentre real‐world evaluation of the Haematuria Cancer Risk Score to risk‐stratify the detection of bladder cancer in patients referred from primary care
Source: BJUI Compass. 2026 May 29;7(6):e70233. doi: 10.1002/bco2.70233 (PMC13239896; doi:10.1002/bco2.70233)
Supplement: Supplementary file 1 — Table S1: Grade and stage of bladder tumours. [file BCO2-7-e70233-s001.docx]

**Supplementary Table 1: Grade and stage of bladder tumours**

| **Stage and grade of bladder tumour** | **Total number** | **Referred with NVH** | **Referred with VH** | **Referred with other features** | |
| --- | --- | --- | --- | --- | --- |
| **CIS** | **2** | 2 (100%) | 0 | 0 | |
| **Low grade NMIBC** | **69** | 3 (4%) | 62 (90%) | 4 (6%) | |
| *G1 pTa* | *11* |  |  |  |  |
| *G2 (low) pTa* | *58* |  |  |  |  |
| **High grade NMIBC** | **98** | 7 (7%) | 86 (88%) | 5 (5%) | |
| *G2 (high) pTa* | *6* |  |  |  |  |
| *G2 (low) pT1* | *5* |  |  |  |  |
| *G2 (high) pT1* | *6* |  |  |  |  |
| *G3 pTa* | *28* |  |  |  |  |
| *G3 pT1* | *52* |  |  |  |  |
| **Localised MIBC** | **24** | 1 (4%) | 22 (92%) | 1 (4%) | |
| *T2 N0 M0 TCC* | *23* |  |  |  |  |
| *T2 N0 M0 small cell carcinoma* | *1* |  |  |  |  |
| **Metastatic MIBC** | **9** | 1 (11%) | 8 (89%) | 0 | |
| *T2 N2 M0 TCC* | *1* |  |  |  |  |
| *T2 N3 M0 TCC* | *1* |  |  |  |  |
| *T2 N0 M1 TCC* | *1* |  |  |  |  |
| *T2 NX MX TCC* | *1* |  |  |  |  |
| *T3 N0 M0 TCC* | *1* |  |  |  |  |
| *T3 N0 M0 squamous cell carcinoma* | *1* |  |  |  |  |
| *T4 N2 M1 TCC* | *1* |  |  |  |  |
| *T4 N2 M1 TCC with sarcomatoid/neuroendocrine/small cell differentiation* | *1* |  |  |  |  |
| *T4 N3 M0 squamous cell carcinoma* | *1* |  |  |  |  |
| *CIS carcinoma in-situ, NMIBC non-muscle invasive bladder cancer, MIBC muscle-invasive bladder cancer* | | | | |  |
